# Convergence of TGFβ and BMP signaling in regulating human bone marrow stromal cell differentiation

**DOI:** 10.1038/s41598-019-41543-0

**Published:** 2019-03-21

**Authors:** Mona Elsafadi, Tasneem Shinwari, Sami Al-Malki, Muthurangan Manikandan, Amer Mahmood, Abdullah Aldahmash, Musaad Alfayez, Moustapha Kassem, Nehad M. Alajez

**Affiliations:** 1 Stem Cell Unit, Department of Anatomy, College of Medicine, King Saud University, Riyadh, Saudi Arabia; 2 College of Agriculture, King Saud University, Riyadh, Saudi Arabia; 3 Prince Naif Health Research Center, King Saud University, Riyadh, 11461 Saudi Arabia; 4 KMEB, Department of Endocrinology, University Hospital of Odense and University of Southern Denmark, Odense, Denmark; 5 Cancer Research Center, Qatar Biomedical Research Institute, Hamad Bin Khalifa University (HBKU), Qatar Foundation, PO Box 34110 Doha, Qatar; 6 College of health & life sciences, Hamad Bin Khalifa University (HBKU), Qatar Foundation, Doha, Qatar

**Keywords:** Cell biology, Stem cells

## Abstract

Targeting regulatory signaling pathways that control human bone marrow stromal (skeletal or mesenchymal) stem cell (hBMSC) differentiation and lineage fate determination is gaining momentum in the regenerative medicine field. Therefore, to identify the central regulatory mechanism of osteoblast differentiation of hBMSCs, the molecular phenotypes of two clonal hBMSC lines exhibiting opposite *in vivo* phenotypes, namely, bone forming (hBMSC^+bone^) and non-bone forming (hBMSC^−Bone^) cells, were studied. Global transcriptome analysis revealed significant downregulation of several TGFβ responsive genes, namely, TAGLN, TMP1, ACTA2, TGFβ2, SMAD6, SMAD9, BMP2, and BMP4 in hBMSC^−Bone^ cells and upregulation on SERPINB2 and NOG. Transcriptomic data was associated with marked reduction in SMAD2 protein phosphorylation, which thereby implies the inactivation of TGFβ and BMP signaling in those cells. Concordantly, activation of TGFβ signaling in hBMSC^−Bone^ cells using either recombinant TGFβ1 protein or knockdown of *SERPINB*2 TGFβ-responsive gene partially restored their osteoblastic differentiation potential. Similarly, the activation of BMP signaling using exogenous BMP4 or *via* siRNA-mediated knockdown of NOG partially restored the differentiation phenotype of hBMSC^−Bone^ cells. Concordantly, recombinant NOG impaired *ex vivo* osteoblastic differentiation of hBMSC^+Bone^ cells, which was associated with SERBINB2 upregulation. Our data suggests the existence of reciprocal relationship between TGFB and BMP signaling that regulates hBMSC lineage commitment and differentiation, whilst provide a plausible strategy for generating osteoblastic committed cells from hBMSCs for clinical applications.

## Introduction

Human bone marrow-derived stromal (skeletal or mesenchymal) stem cells (hBMSC) exhibit the potential to differentiate into various mesodermal cells including osteoblasts, adipocytes, and chondrocytes^1^. These have all been employed in regenerative medicine protocols for treating skeletal diseases e.g. non-healed fractures and the repair of bone defects^2^. However, cultured hBMSC cells exhibit functional and molecular heterogeneity with respect to differentiation capacity and bone formation potential^3,4^. This may explain the variability in the results obtained from hBMSC-based therapies^5^. One possible approach to enhance the therapeutic efficacy of hBMSC in bone regeneration protocols is to employ osteoblast-committed progenitors. Moreover, in certain disease conditions such as osteoporosis, for example, the impairment of osteoblast differentiation of hBMSC occurs, thereby necessitating the *in vivo* enhancement of the bone forming capacity of hBMSC^6^. However, this requires the identification of the signaling pathways and molecules that regulate hBMSC commitment into the osteoblastic lineage^7,8^.

We have previously employed global transcriptomics and proteomic approaches in order to identify the molecules and signaling pathways regulating hBMSC lineage specific differentiation based on studying the *in vitro* differentiation dynamics of hBMSC^3,9–11^. Several follow up studies led to the identification of factors that are relevant for *in vitro* osteoblast differentiation and *in vivo* bone formation^12,13^. Whilst this approach is both useful and hypothesis-generating, it requires extensive and time-consuming screening. In the current study, we performed reverse molecular phenotyping which is currently used in precision medicine. In this approach, the *in vivo* phenotype is interrogated based on molecular phenotyping in order to identify the signaling pathways which are to be targeted in individualized therapy. Using a similar approach, we tested the possibility of identifying those signaling pathways relevant for *in vivo* bone formation based on the ability of hBMSC to form bone *in vivo*^14^. We employed two previously established hBMSC lines derived from telomerase-immortalized hBMSCs (hBMSC-TERT) that exhibited either ectopic bone forming or non-bone forming phenotype when implanted *in vivo* into immunodeficient mice^3,15^. Employing whole transcriptome profiling comparing these two hBMSC lines, we identified the molecular signature and signaling pathways associated with the bone-forming phenotype. Most importantly, our data suggest the convergence of TGFβ- and BMP4-signaling pathways during osteoblastic lineage commitment of hBMSC.

## Materials and Methods

### Ethics statement

This study did not involve human or animal subjects, therefore ethical approval is not required.

### Cell culture

We employed the hMSC-TERT cell line which was created from primary normal human MSC by overexpressing human telomerase reverse transcriptase gene (hTERT)^16^. The hMSC-TERT cells have been extensively characterized and they exhibited similar cellular responses and molecular phenotype to primary hBMSC^17^. For ease, we will refer to this cell line as ‘hBMSC’ for the remaining part of this manuscript. In the current experiment, we employed two sub-clones of high bone-forming cells (hBMSC^+Bone^) and low bone-forming cells (hBMSC^−Bone^) which were derived from early-passage hBMSC-TERT cells [with a population doubling level of (PDL) 77] as well as from late-passage hBMSC-TERT cells (PDL = 233), respectively, as previously described^3^. The cells were cultured in Dulbecco’s Modified Eagle Medium (DMEM) supplemented with D-glucose 4500 mg/L, 4 mM L-Glutamine, 110 mg/L Sodium Pyruvate, 10% Fetal Bovine Serum (FBS), 1x penicillin–streptomycin (Pen-strep), and non-essential amino acids (all purchased from Thermo Fisher Scientific, Waltham, MA), at 37 °C in a humidified atmosphere containing 5% CO2.

### siRNA-mediated transfection of hMSC

For transfection experiments, hBMSC cells in logarithmic growth phase were reverse-transfected with Silencer Select Pre-designed and Validated SERPINB2-siRNA (25 nM) (Ambion ID: s10016, s10017, and s10018, Cat. No. 4392420, Thermo Fisher Scientific Life Sciences, USA), or NOG-siRNA (25 nM) (Ambion ID: s534108, Cat. No. 4392420) using Lipofectamine 2000 Reagent (Invitrogen), plus serum-free Opti-MEM I medium (Thermo Fisher Scientific, Waltham, MA) as per the manufacturer’s recommendations. On day 3 of transfection, the cells were induced into osteoblast (OS) or adipocyte (AD) media.

### *In vitro* osteoblast differentiation

Cells were grown in standard DMEM growth medium in 6-well plates at 0.3 × 10^6^ cells/ml. When a 70–80% cell confluence was reached, the cells were cultured in DMEM supplemented with an osteoblast induction mixture containing 10% FBS, 1% Pen-strep, 50 μg/ml L-ascorbic acid (Wako Chemicals, Neuss, Germany), 10 mM β-glycerophosphate (Sigma), 10 nM calcitriol (1α,25-dihydroxy vitamin D3; Sigma), and 10 nM dexamethasone (Sigma). The media was replaced 3 times per week.

### *In vitro* adipocyte differentiation

Cells were grown in standard DMEM growth medium in 6-well plates at 0.3 × 10^6^ cells/ml. When a 90–100% cell confluence was reached, the cells were cultured in DMEM supplemented with adipogenic induction mixture containing 10% FBS, 10% Horse Serum (Sigma-Aldrich, St. Louis, MO), 1% Pen-strep, 100 nM dexamethasone, 0.45 mM isobutyl methyl xanthine^18^ (Sigma, US), 3 μg/mL insulin (Sigma, US), and 1 μM Rosiglitazone^19^ (Novo Nordisk, Bagsvaerd, Denmark). The media used was replaced 3 times per week.

### Cytochemical staining

#### Alizarin Red S staining for mineralized matrix

The cell layer was washed with PBS, and then fixed with 4% paraformaldehyde for 15 minutes at room temperature. After removing the fixative, the cell layer was rinsed in distilled water and stained with 2% Alizarin Red S Staining Kit (ScienCell Research Laboratories,

Carlsbad, CA, Cat. No. 0223) for 20–30 minutes at room temperature. Any excess dye was washed off with water. For quantifying the Alizarin Red S staining, the Alizarin Red S dye was eluted in 800 µl of acetic acid and then incubated in each well for 30 minutes at room temperature as described before^20^ and measured using the Biotek™ Epoch™ Microplate Spectrophotometer (BioTek™ Instruments Inc., USA) at 405 nm.

### Quantitative ALP activity

To quantify ALP activity in hBMSC before and after OS differentiation, we used the BioVision ALP activity colorimetric assay kit (Biovision Inc., Milpitas, CA) with some modifications. Cells were cultured in 24-well plates under normal conditions; then, on the day of analysis, wells were rinsed once with PBS and were fixed using 3.7% formaldehyde in 90% ethanol for 30 seconds at room temperature. Subsequently, the fixative was removed, and 50 *μ*L of pNPP solution was added to each well and the cells were next incubated for 1 hour in the dark at room temperature. The reaction was subsequently stopped by adding 20 *μ*L stop solution and gently shaking the plate. The OD was then measured at 405 nm.

### OsteoImage mineralization assay

The *in vitro* formed mineralized matrix was quantified using the OsteoImage™ Mineralization Assay Kit (LONZA, USA, Cat. No. PA-1503). After this, the culture media was removed and the cells were washed once with PBS and then fixed with 70% cold ethanol for 20 minutes. The appropriate amount (as per the manufacturer’s recommendations) of diluted staining reagent was added, and the plates were incubated in the dark for 30 minutes at room temperature. The cells were then washed and staining quantitation was performed using a fluorescent plate reader (SpectraMax M5 Molecular Devices, Sunnyvale, CA) at 492/520 excitation emission wavelengths.

### Oil red-O staining for lipid droplets

Mature adipocytes filled with cytoplasmic lipid droplets were visualized by staining with Oil Red-O. After washing with PBS, the cells were fixed in 4% formaldehyde for 10 minutes at room temperature, then rinsed once with 3% isopropanol, and stained for 1 hr at room temperature with filtered Oil Red-O staining solution (prepared by dissolving 0.5 g Oil Red-O powder in 60% isopropanol). To quantify the mature adipocytes that were formed, Oil Red O stain was eluted by adding 100% isopropanol to each well. The color intensity was then measured using Biotek™ Epoch™ Microplate Spectrophotometer (BioTek Instruments Inc., Winooski, VT) spectrophotometer at 510 nm.

### Nile red fluorescence determination and quantification of mature adipocytes

A stock solution of Nile red (1 mg/ml) in DMSO was prepared and stored at −20 °C protected from light exposure. Staining was performed on fixed cells using 4% paraformaldehyde (Sigma) for 15 minutes. Cultured undifferentiated and differentiated cells were washed once with PBS. The dye was then added directly to the cells (5 *μ*g/ml in PBS), and the cells were incubated for 10 min at RT. Fluorescent signals were measured using the SpectraMax/M5 fluorescence spectrophotometer plate reader (Molecular Devices Co., Sunnyvale, CA) using the bottom well-scan mode where nine readings were taken per well using an excitation level of 485 nm and an emission level of 572 nm.

### Cell proliferation assays

Cell viability was measured using the alamarBlue assay according to the manufacturer’s recommendations (Thermo Fisher Scientific, Waltham, MA). In brief, 10 μl of alamarBlue substrate was added to cultured cells in 96-well plates and the plates were incubated in the dark at 37 °C for 1 h. The reading was subsequently taken using fluorescent mode (Ex 530 nm/Em 590 nm) using the BioTek™ Synergy II microplate reader (BioTek Inc., Winooski, VT, USA).

### Western blot analysis

Cells were lysed using RIPA buffer (Thermo Fisher Scientific, Waltham, MA) and soluble proteins were immunoblotted using P-SMAD2 (Cell Signaling Technology, Danvers, MA, Cat no. 9523, diluted 1:500) and anti-β-ACTIN (Sigma-Aldrich, St. Louis, MO, A3854, diluted according to a ratio of 1:10,000). Reactivity was detected with horseradish peroxidase-conjugated secondary antibodies (Santa-Cruz Biotechnology, Inc., Dallas, TX) and Clarity™ western ECL substrate (Bio-Rad) for chemiluminescence using C-Digit Blot Scanner (Li-Cor Bioscience, Lincoln, NE).

### DNA microarray global gene expression profiling

Total RNA was extracted using PureLink RNA mini isolation kit (by Ambion Life Technologies, Carlsbad, CA, Cat No: 12183018 A) as recommended by the manufacturer. One hundred and fifty nanograms of total RNA were labeled and then hybridized to the Agilent Human SurePrint G3 Human GE 8 × 60 k microarray chip (Agilent Technologies, Santa Clara, CA). All microarray experiments were conducted at the Microarray Core Facility (Stem Cell Unit, King Saud University College of Medicine). Normalization and data analyses were conducted using GeneSpring GX software (Agilent Technologies). Pathway analysis was conducted using the Single Experiment Pathway analysis feature in GeneSpring 12.0 (Agilent Technologies) as previously described^21^. A two fold cutoff with P < 0.02 was used.

### Quantitative real time PCR (qRT-PCR)

Total RNA was extracted using PureLink kit (Ambion Life Technologies, Carlsbad, CA, Cat No: 12183018A) as recommended by the manufacturer. Total RNA was quantified by using the Nanodrop™ spectrophotometer (Nanodrop 2000, Thermo Fisher Scientific, Inc., Waltham, MA). Complementary DNA (cDNA) was synthesized from 1 μg of the RNA using a High Capacity cDNA Reverse Transcription kit (Applied Biosystem, USA) and Labnet Multigene themocycler (Labnet International Inc., Edison, NJ) according to the manufacturer’s instructions. Relative levels of mRNA were determined from cDNA using Power SYBR Green PCR kit or the TaqMan Universal master Mix II with no UNG, both from Applied Biosystems (Applied Biosystems, Foster City, CA) according to the manufacturer’s instructions. Following normalization to the reference gene GAPDH, the quantification of gene expression was carried out by using a comparative Ct method where ΔCT is the difference between the CT values of the target and the reference gene. The primers that were employed are listed in supplementary Tables 1.

### Statistical analysis

All of the results were presented as the mean and standard deviation (SD) of at least 3 independent experiments. A Student’s t-test was used for testing the differences between groups. *P*-values < 0.05 was considered statistically significant.

## Results

### Molecular heterogeneity of bone-and non-bone-forming hBMSC clones

**Figure 1:**
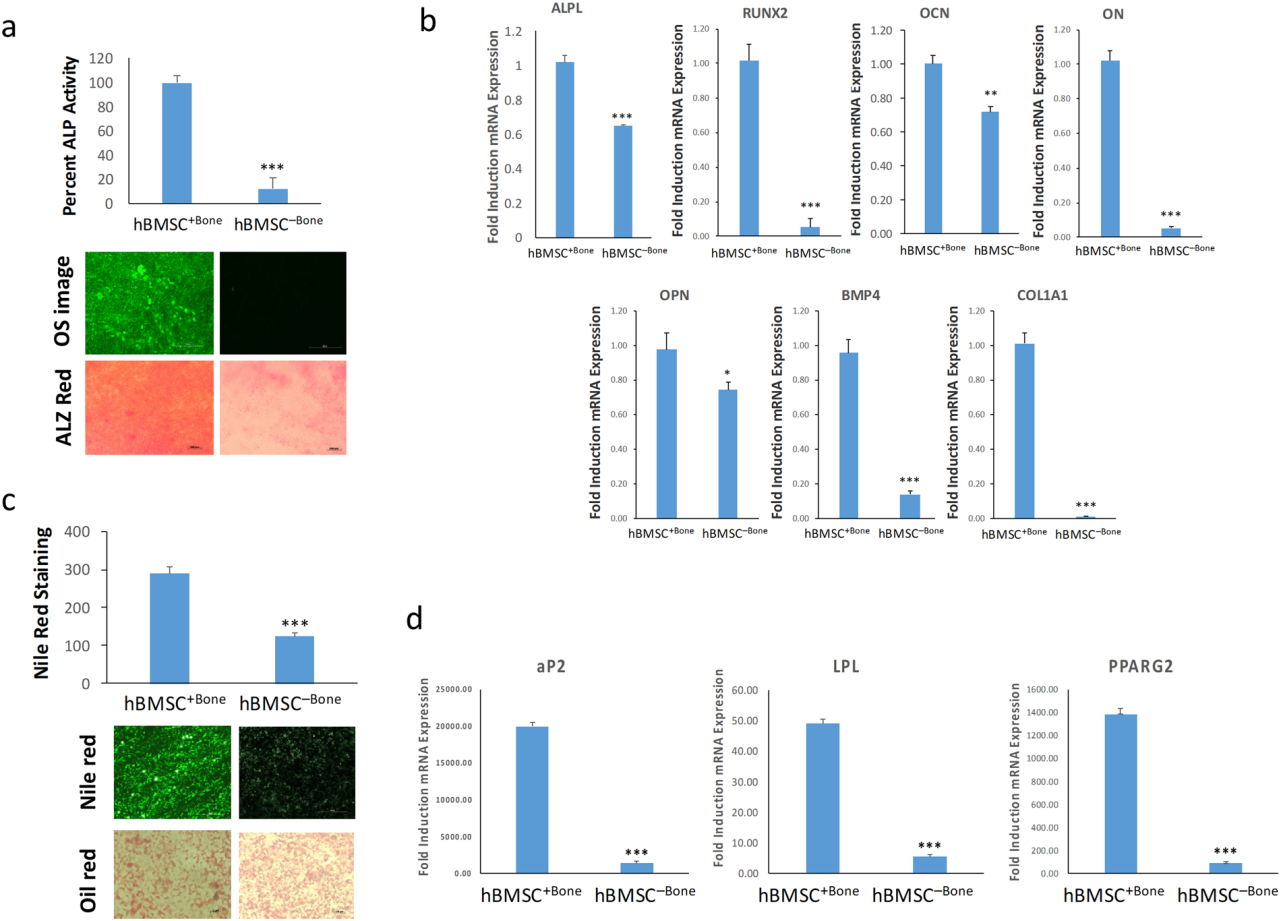
Functional heterogeneity of bone- and non-bone- forming hBMSC clones. **(a)** Quantification of percent ALP activity on day 14. Data is presented as the means ± SD of three independent experiments; *n* = 6; ***p < 0.0005. The upper image panel shows OsteoImage™ staining, while the lower panel shows Alizarin Red S staining. **(b)** qRT-PCR quantification of ALPL, RUNX2, OCN, ON, OPN, BMP4, and COL1A1 osteoblast markers under osteoblastic induction conditions. The expression of each target gene was normalized to *GAPDH*. Data are presented as mean ± SD from three independent experiments, *n* = 9; *p < 0.05; **p < 0.005, ***p < 0.0005. **(c)** Nile red quantification of mature adipocytes on day 7 post adipocyte induction of the indicated hBMSC clone. Data are presented as mean ± SD, *n* = 9 from three independent experiments. ***p < 0.0005. Upper panel shows Nile red staining of mature oil filled adipocytes, while the lower panel shows oil red O staining for adipocyte (20× magnification). **(d)** qRT-PCR quantification for aP2, LPL and *PPARγ2*. The expression of each target gene was normalized to *GAPDH*. Data is presented as the means ± SD from three independent experiments, *n* = 9; ***p < 0.0005.

We previously derived two clonal hBMSC lines with bone-forming (hBMSC + Bone) or non-bone forming (hBMSC−Bone) properties. The clonal lines were derived from the parental hBMSC-TERT cell line)^3^. As shown in Fig. 1a, hBMSC−Bone exhibited low osteoblastic (OB) differentiation potential when compared to hBMSC^+Bone^ as evidenced by decreased ALP activity (Fig. 1a, upper panel) as well as decreased extracellular mineralized matrix formation (Fig. 1a, lower panel). The expression of the osteoblastic lineage gene markers: alkaline phosphatase (ALPL), runt-related transcription factor 2 (RUNX2), osteocalcin (OCN), osteonectin (ON), osteopontin (OPN), bone morphogenic protein 4 (BMP4), and collagen-1A1 (COL1A1) was also decreased (see Fig. 1b). Similarly, hBMSC−Bone showed low *in vitro* adipocytic (AD) differentiation potential as evidenced by the decreased formation of mature lipid-filled adipocytes (Fig. 1c,d) as well as the reduced expression of the adipocyte lineage gene markers: adipocyte protein 2 (aP2), lipoprotein lipase (LPL), and peroxisome proliferator-activated receptor gamma 2 (PPARg2) (Fig. 1e). hBMSC^+Bone^ cells exhibited enhanced differentiation potential into osteoblastic and adipocytic cells versus hBMSC-Bone, which has limited differentiation capacity.

### Impaired TGFβ signaling pathway in hBMSC^−Bone^

**Figure 2:**
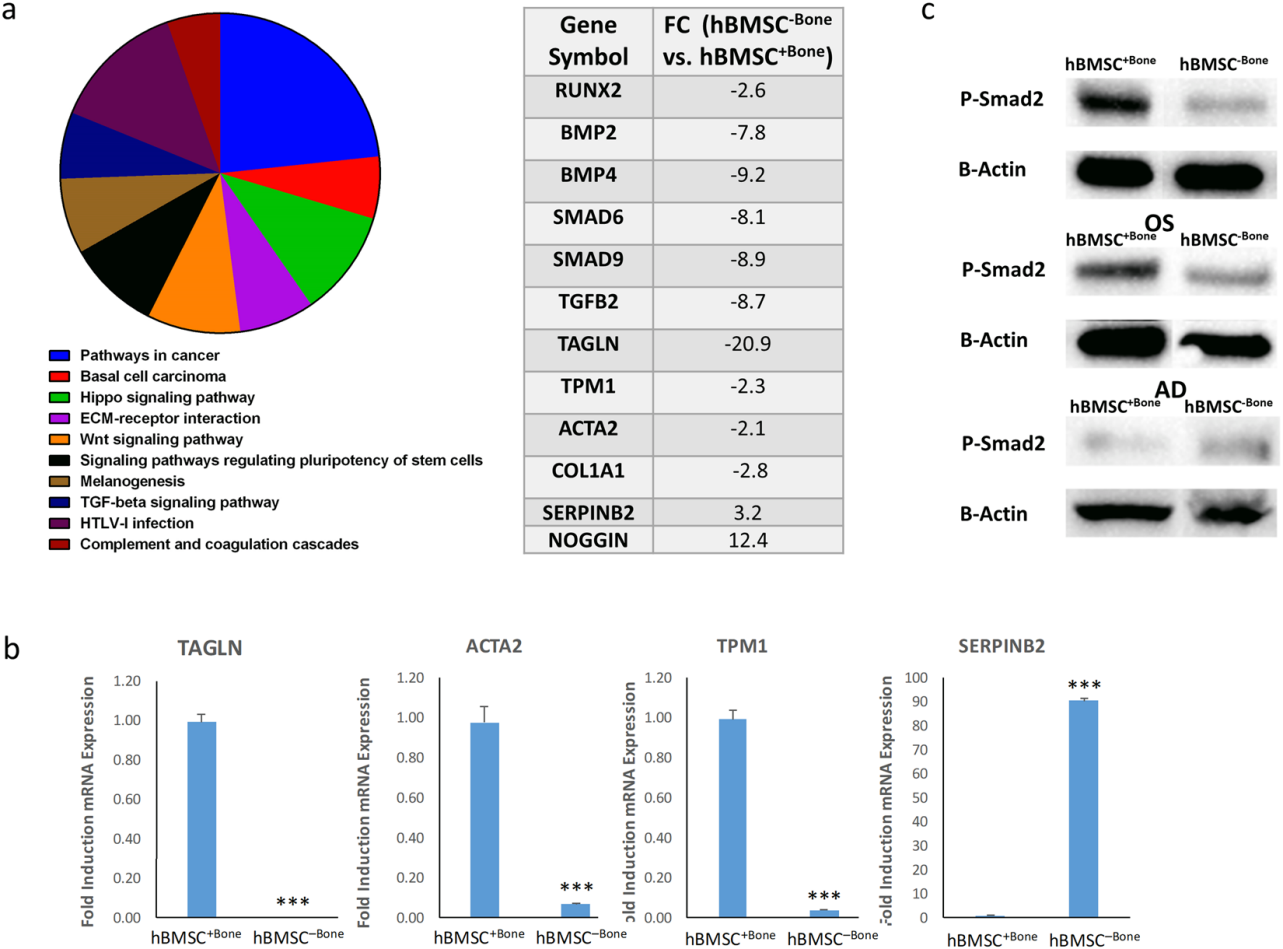
Impaired TGFβ signaling in hBMSC^−Bone^ cells. (**a)** Pie chart illustrating the distribution of the top 10 KEGG pathways in the downregualted genes. The pie size corresponds to the number of matched entities. List of TGFβ responsive genes, which were differentially expressed in hBMSC^−Bone^ *vs*. hBMSC^+Bone^ as revealed by whole genome microarray profiling is shown. **(b)** qRT-PCR validation for the expression of a panel of TGFβ responsive genes (TAGLN, ACTA2, TPM1, and SERPINB2) *in* hBMSC^−Bone^ compared to hBMSC^+Bone^ cells. Expression of each target gene was normalized to GAPDH. Data is shown as the mean ± SD from three independent experiments, ***p < 0.0005. **(c)** Western blotting for P-SMAD2 in hBMSC^−Bone^ compared to hBMSC^+Bone^ cells (upper panel), whereas B-Actin (ACTB, lower panel) was used as a loading control. Phosphorylation of SMAD2 is also shown during the osteogenic and adipogenic differentiation of both cell lines.

We compared the whole transcriptome using global gene expression profiling of hBMSC^+Bone^ and hBMSC^−Bone^ to identify the molecular signature that was predictive of functional divergence. The top ten significantly enriched KEGG pathways in the downregulated genes in hBMSC^−Bone^ is illustrated as pie chart in Fig. 2a. Interestingly, several TGFβ-responsive genes were dysregulated in hBMSC^−Bone^ compared with hBMSC^+Bone^ (Fig. 2a) including *RUNX2*, *BMP2*, *BMP4*, *SMAD6*, *SMAD9*, TGFβ*2*, *TAGLN*, *TPM1*, *ACTA2*, *COL1A1*, *SERPINB2*, and *NOG*, suggesting the suppression of the TGFβ signaling pathway in hBMSC^−Bone^. Validation of the microarray data using qRT-PCR revealed good concordance between the microarray data and qRT-PCR for a selected panel of TGFβ responsive genes including: *TAGLN*, *ACTA2*, *TPM1*, and *SERPINB2* (see Fig. 2b). Our previous data demonstrated inverse correlation between SERPINB2 upregulation and TGFB activation^22^. Furthermore, Western blot analysis of phosphorylated SMAD2 (p-SMAD2) revealed a marked reduction in p-SMAD2 in hBMSC^−Bone^ *vs*. hBMSC^+Bone^ at baseline (Fig. 2c, upper panel), on day 10 during *in vitro* osteoblastic (Fig. 2c, middle panel), as well as adipocytic (Fig. 2c, lower panel) differentiation. Taken together, those data demonstrated impaired TGFβ signaling in the hBMSC^−Bone^ line.

### Exogenous TGFβ1 promotes osteogenic and adipogenic differentiation of hBMSC^−Bone^ cells

**Figure 3:**
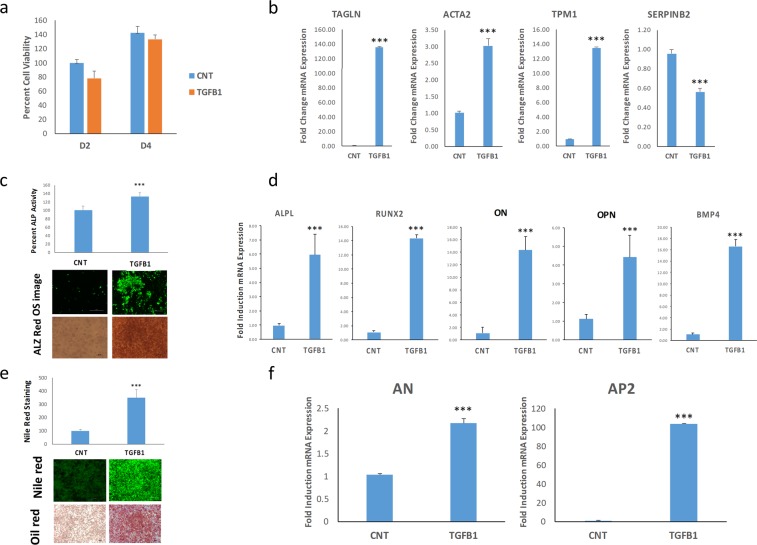
Exogenous TGFβ1 stimulus promotes osteogenic and adipogenic differentiation of hBMSC^−Bone^ cells. (**a**) Quantitative cell viability for hBMSC^−Bone^ cells on days 2 and 4 in the presence or absence of TGFβ1 treatment (10 ng/ml). **(b)** qRT-PCR quantification of TAGLN, ACTA2, TPM1, and SERPINB2 TGFβ responsive genes in hBMSC^−Bone^ cells in the presence or absence of TGFβ1 treatment (10 ng/ml). The expression of each target gene was normalized to GAPDH. Data are presented as mean ± SD from three independent experiments, ***p < 0.0005. **(c)** Percentage ALP activity in hBMSC^−Bone^ in the presence or absence of TGFB1 on day 14. Data is presented as the means ± SD from three independent experiments; *n* = 6; ***p < 0.0005. The upper image panel shows OsteoImage™ staining (20x magnification), while the lower panel shows Alizarin Red S staining. **(d)** qRT-PCR quantification for ALPL, RUNX2, ON, OPN, and BMP4 osteogenic markers performed on hBMSC^−Bone^ cells exposed to osteogenic induction medium in the presence or absence of TGFβ1. The expression of each target gene was normalized to GAPDH. Data are presented as the means ± SD from three independent experiments, *n* = 9; b***p < 0.0005. **(e)** Nile red quantification of hBMSC^−Bone^ under the indicated treatment conditions on day 7 post adipocyte induction. Data are presented as the means ± SD, *n* = 9 from three independent experiments; ***p < 0.0005. Upper images shows fluorescence Nile red staining of mature oil filled adipocytes (20× magnification), while the lower panel shows oil red O staining of adipocytes (20× magnification). **(f)** qRT-PCR quantification for AN and AP2 mRNA. Expression of each target gene was normalized to GAPDH. Data is presented as the means ± SD from three independent experiments, *n* = 9; ***p < 0.0005.

We subsequently assessed the effect of TGFβ1 (10 ng/ml) treatment on hBMSC^−Bone^ cell proliferation and differentiation into osteoblasts and adipocytes. The hBMSC^−Bone^ cells exhibited no changes in cell proliferation or viability when treated with TGFβ1, (Fig. 3a); however, TGFB1 treatment led to upregulation of a number of TGFβ-responsive genes (TGALN, ACTA2, and TPM1) and the downregulation of *SERPINB2* (Fig. 3b). ALP activity and the quantification of formed mineralized matrix revealed significant increase in the osteoblastic differentiation of hBMSC^−Bone^ in response to TGFβ1 treatment (Fig. 1c) and was corroborated by the increased gene expression of the osteoblastic markers: ALPL, RUNX2, ON, OSP, and BMP4 (Fig. 3d). Similarly, qualitative and quantitative Nile red staining of mature adipocytes revealed enhanced adipogenesis in response to TGFβ1 treatment (Fig. 3e,f). The data we have generated, therefore, supports a role for TGFB signaling in the regulation of both osteoblast and adipocyte differentiation of hBMSC^−Bone^ cells, where activation of TGFβ signaling in hBMSC^−Bone^ cells using recombinant TGFβ1 protein as able to rescue their osteoblastic differentiation phenotype.

### Silencing SERPINB2 promotes osteoblastic and adipocytic differentiation of hBMSC^−Bone^ cells

**Figure 4:**
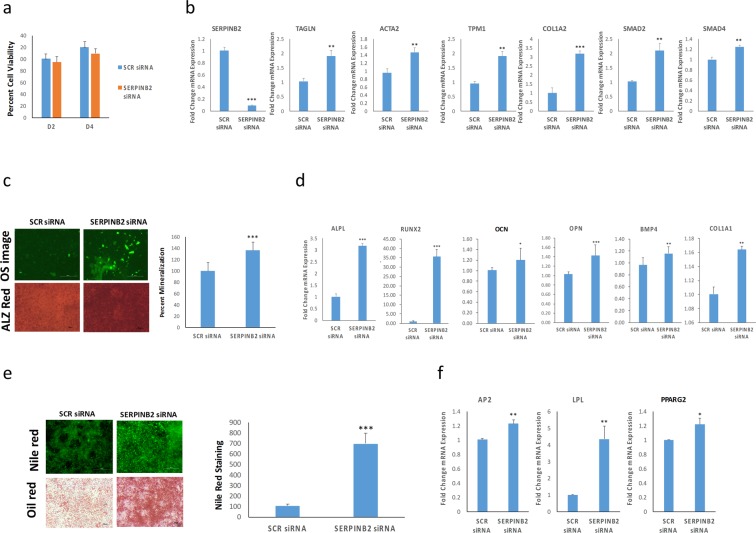
Downregulation of SERPINB2 promotes osteoblastic and adipocytic differentiation of hBMSC^−Bone^ cells. (**a**) Alamarblue quantification for cell viability of hBMSC^−Bone^ cells transfected with scramble-siRNA (SCR) or SERPINB2-siRNA on days 2 and 4. **(b)** qRT-PCR for SERPINB2, TAGLN, ACTA2, TPM1, COL1A2, SMAD2 and SMAD4 TGFβ responsive genes in SERPINB2-depleted *vs*. scramble-siRNA (SCR) control cells. The expression of each target gene was normalized to GAPDH. Data are presented as mean ± SD from three independent experiments; **p < 0.005, ***p < 0.0005. **(c)** Shows the OsteoImage™ staining (20× magnification) in differentiated hBMSC^−Bone^ cells post SERPINB2 knockdown compared to scramble-siRNA transfected control cells. The lower panel shows Alizarin Red S staining. Quantification of mineralized matrix formation under different treatments is shown on the right panel. Data are presented as mean mineralization ± SD from three independent experiments, *n* = 9; ***p < 0.0005. **(d)** qRT-PCR quantification of ALPL, RUNX2, OCN, OPN, BMP4, and COL1A1 osteogenic markers mRNA expression in SERPINB2-depleted *vs*. scramble-siRNA (SCR) transfected hBMSC^−Bone^ cells under osteogenic induction conditions. The expression of each target gene was normalized to GAPDH. Data are presented as the means ± SD from three independent experiments, *n* = 9; *p < 0.05; **p < 0.005, ***p < 0.0005. **(e)** Nile red staining of mature oil filled adipocytes (20× magnification) in hBMSC^−Bone^ cells on day 7 post adipocytic differentiation. Oil red O staining is shown in the lower panel (20× magnification). The right panel shows quantification of Nile red staining, ***p < 0.0005. **(f)** qRT-PCR quantification for aP2, LPL and PPARG2 adipogenic markers. Expression of each target gene was normalized to GAPDH. Data are presented as means ± SD from three independent experiments, *n* = 9; *p < 0.05; **p < 0.005.

As shown in Fig. 2a, we observed elevated gene expression levels of SERPINB2 (3.2 FC), a TGFB-responsive gene, in the hBMSC^−Bone^ cells. We have previously reported a negative regulatory role for SERPINB2 in hBMSC differentiation^22^. Thus, we employed a loss-of-function approach to determine the role of SERPINB2 in hBMSC^−Bone^ biology. The siRNA-mediated depletion of *SERPINB2* had no effect on cell viability (Fig. 4a), while it led to significant increase in the expression of TGFβ responsive genes, such as TAGLN, ACTA2, TPM1, COL1A2, SMAD2, and SMAD4 (Fig. 4a). In addition, SERPINB2-depleted hBMSC^−Bone^ cells exhibited enhanced osteoblastic differentiation potential as demonstrated by increased qualitative and quantitative mineralized matrix formation (Fig. 4c), and associated with upregulation of the osteoblastic gene markers: ALPL, RUNX2, OCN, OPN, BMP4, and COL1A1 (Fig. 4d). Similarly, SERPINB2 depletion during adipogenesis led enhanced adipocytic differentiation characterized by the increase in the number of Nile red positive mature adipocytes (Fig. 4e) as well as the upregulation of adipocyte gene markers: AP2, LPL, and PPARG2 (Fig. 4f). Therefore, activation of TGFβ signaling in hBMSC^−Bone^ cells using siRNA-mediated knockdown of *SERPINB2* partially restored their osteoblastic differentiation potential.

### Gene expression profiling of SERPINB2-depleted hBMSC^−Bone^ cells

**Figure 5:**
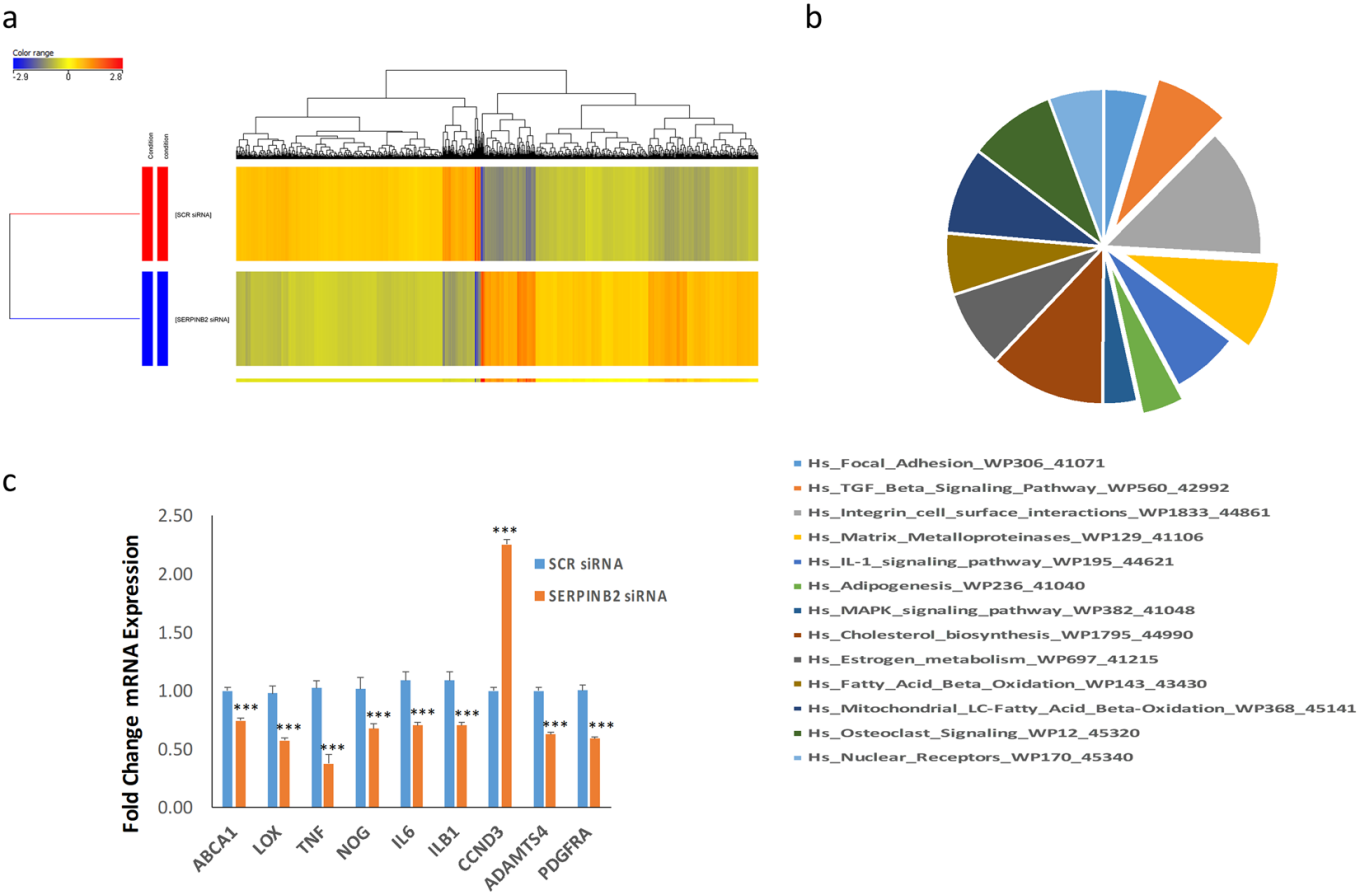
Gene expression profiling of SERPINB2-depleted hBMSC^−Bone^ cells under osteogenic conditions. (**a**) Hierarchical clustering of SERPINB2-depleted hBMSC^−Bone^ cells compared to scramble-siRNA transfected control cells, based on differentially expressed mRNA transcripts. The expression level of each gene in each condition is depicted according to the color scale shown. **(b)** Pie chart illustrating the distribution of top pathway designations for the de-regulated genes in SERPINB2-depleted hBMSC^−Bone^ cells. **(c)** The expression levels of selected genes from the microarray data was validated using qRT-PCR in SERPINB2-depleted compared to Scrambled siRNA-transfected control hBMSC^−Bone^. Data are presented as the means ± SD from two independent experiments, *n* = 6; ***p < 0.0005.

Given the observed effects of SERPINB2-depletion on rescuing osteoblastic and adipocytic differentiation of hBMSC^−Bone^ cells, we sought to determine the underlying molecular mechanisms linking SERPINB2 to osteoblastic and adipocytic differentiation in hBMSC^−Bone^ cells. Hence, we performed global gene expression profiling on SERPINB2-depleted hBMSC^−Bone^ compared to scrambled-transfected control cells. Hierarchical clustering based on differentially expressed transcripts revealed distinct clustering of the two groups (Fig. 5a). We identified 480 up-regulated and 423 down-regulated genes in SERPINB2-depleted hBMSC^−Bone^ cells (2.0 FC, p < 0.05; Supplementary Table 2). Pathway analysis was performed on the differentially expressed mRNA transcripts revealing significant enrichment in several signaling pathways including focal adhesion, TGFβ signaling, adipogenesis, matrix metalloproteinases, MAPK, and osteoclast signaling (Fig. 5b). Good concordance was observed between the microarray data and qRT-PCR validation of the regulation of a selected number of differentially expressed genes (Fig. 5c). Therefore, or global transcriptome analysis revealed significant restoration of TGFβ signaling pathway in SERPINB2-depleted hBMSC^−Bone^ cells.

### NOG-depleted hBMSC^−Bone^ cells exhibited enhanced osteoblastic and adipocytic differentiation

**Figure 6:**
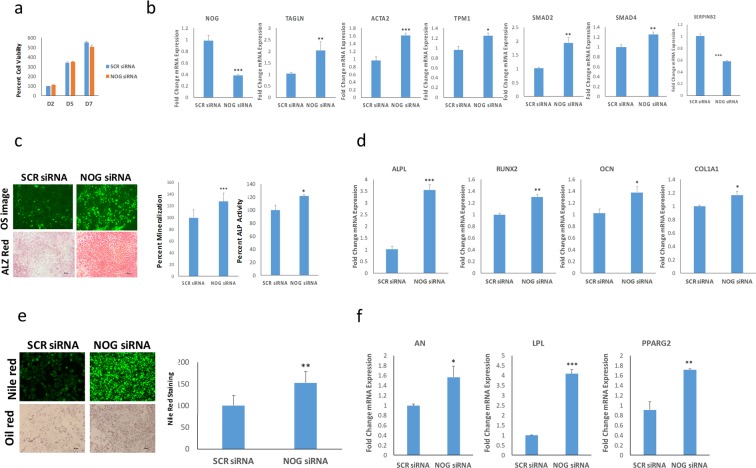
Downregulation of NOG promotes osteoblastic and adipocytic differentiation of hBMSC^−Bone^ cells. (**a**) Quantification of cell viability of hBMSC^−Bone^ cells transfected with NOG-siRNA scramble-siRNA (SCR) measured on days 2, 5, and 7. **(b)** qRT-PCR performed for NOG, TAGLN, ACTA2, TPM1, SMAD2, SMAD4 and SERPINB2 responsive genes in NOG-depleted *vs*. scramble-siRNA (SCR) transfected hBMSC^−Bone^ cells under osteogenic induction conditions. The expression of each target gene was normalized to GAPDH. Data are shown as mean ± SD from three independent experiments, *p < 0.05; **p < 0.005, ***p < 0.0005. **(c)** OsteoImage™ staining (20× magnification) for hBMSC^−Bone^ cells transfected with NOG or control siRNA under osteogenic induction conditions. The lower panel shows Alizarin Red S staining. The quantification of mineralized matrix formation for scramble-siRNA (SCR) and NOG-depleted cells is shown in the left panel, while the quantification of ALP activity under the same experimental conditions is shown in the right panel. Data are presented as relative mean mineralization ± SD from three independent experiments, *n* = 9; *p < 0.05, ***p < 0.0005. **(d)** qRT-PCR quantification of ALPL, RUNX2, OCN, and COL1A1osteogenic markers in scramble-siRNA (SCR) and NOG-depleted hBMSC^−Bone^ cells exposed to osteogenic differentiation medium. The expression of each target gene was normalized to GAPDH. Data are presented as the means ± SD from three independent experiments, *n* = 9; *p < 0.05; **p < 0.005, ***p < 0.0005. **(e)** Nile red staining of hBMSC^−Bone^ cells transfected with scramble-siRNA (SCR) or NOG-specific siRNA, which were then induced into adipocytes for 7 days (20× magnification). The cells were stained using oil red O staining as well. (lower panel, 20× magnification). The right panel shows the quantified fluorescence Nile red staining of mature oil-filled adipocytes. **p < 0.005. **(f)** qRT-PCR quantification for AN, LPL and PPARγ2 adiocytic markers. Expression of each target gene was normalized to GAPDH. Data are presented as the mean ± SD from three independent experiments, *n* = 9; *p < 0.05; **p < 0.005, ***p < 0.0005.

BMP is a signaling pathway that exhibit cross-talk with TGFβ signaling during osteoblastic and adipocytic differentiation of hBMSCs^23,24^. Interestingly, gene expression profiling (Fig. 2a) revealed a marked upregulation of NOG expression (12.4 FC) in hBMSC^−Bone^ cells. To determine the biological relevance of this observation, hBMSC^−Bone^ were transfected with NOG siRNA and were exposed to osteoblastic and adipocytic differentiation induction media. The siRNA-mediated silencing of NOG had no significant effects on cell viability (Fig. 6a), however it led to a significant increase in the expression of several TGFβ responsive genes, including TAGLN, ACTA2, TPM1, SMAD2, and SMAD4 (Fig. 6b). Interestingly, we also observed downregulation of SERPINB2 in NOG-depleted cells. Concordant with TGFB activation, NOG-deficient hBMSC^−Bone^ cells exhibited enhanced osteoblast differentiation as shown by a significant increase in mineralized matrix formation and increased ALP activity (Fig. 6c) as well as an increase in the expression of a number of osteoblastic gene markers: ALPL, RUNX2, OCN, and COL1A1 (Fig. 6d). Similarly, NOG-deficient hBMSC^−Bone^ cells exhibited enhanced adipocytic differentiation shown by the increased number of lipid-filled mature adipocytes (Fig. 6e) and up-regulated expression of AN, LPL and PPARg2 AD gene markers (Fig. 6f). The activation of BMP signaling *via* siRNA-mediated knockdown of NOG partially restored the differentiation phenotype of hBMSC^−Bone^ cells.

### NOG suppresses osteoblastic and adipocytic differentiation of hBMSC^+Bone^ cells

**Figure 7:**
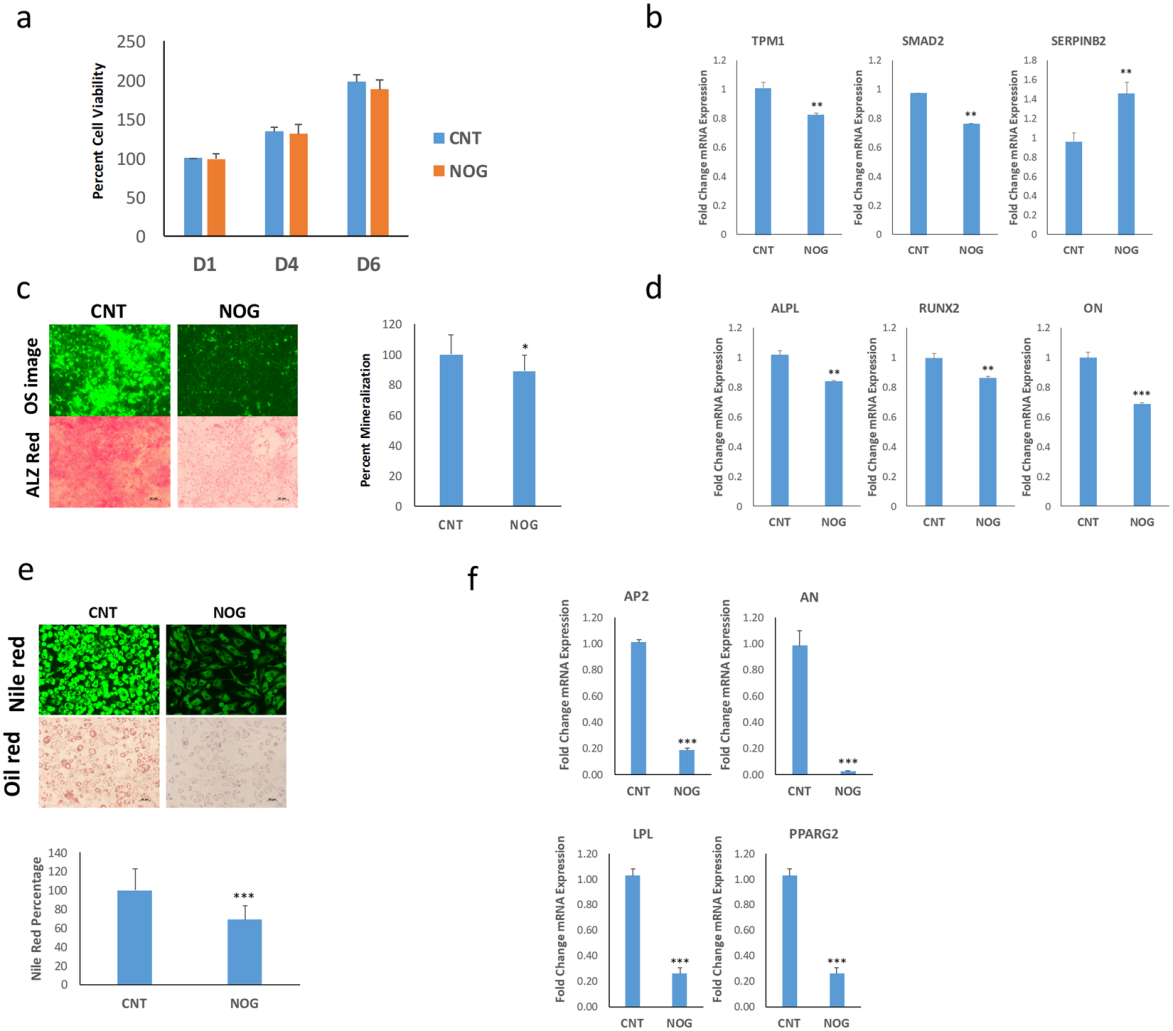
Exogenous NOG suppresses osteoblastic and adipocytic differentiation of hBMSC^+Bone^ cells. (**a**) Quantification of cell viability measured on days 1, 4, and 6 for hBMSC^+Bone^ cells in the presence or absence of recombinant NOG (50 ng/ml). **(b)** qRT-PCR performed for TPM1, SMAD2 and SERPINB2 TGFβ responsive genes in hBMSC^+Bone^ cells in the presence or absence of recombinant NOG (10 ng/ml). The expression of each target gene was normalized to GAPDH. Data are presented as mean ± SD from three independent experiments, **p < 0.005. **(c)** OsteoImage™ staining (20× magnification) of hBMSC^+Bone^ cells which were induced into the osteoblast in the presence or absence of recombinant NOG. The lower panel shows Alizarin Red S staining. The quantification of mineralized matrix formation for vehicle or recombinant NOG-treated hBMSC^+Bone^ cells is shown (right panel). Data are presented as relative mean mineralization ± SD from three independent experiments, *n* = 9; *p < 0.0005. **(d)** qRT-PCR quantification of ALPL, RUNX2, OCN, and COL1A1 osteogenic markers in hBMSC^+Bone^ cells in the presence or absence of recombinant NOG (10 ng/ml) under osteogenic induction conditions. The expression of each target gene was normalized to GAPDH. Data are presented as the means ± SD from three independent experiments, *n* = 9; **p < 0.005, ***p < 0.0005. **(e)** hBMSC^+Bone^ cells were differentiated into adipocytes for 7 days under the indicated experimental conditions. Upper panel shows fluorescence Nile red staining of mature oil filled adipocytes (20× magnification), whilst the lower panel shows Oil red O staining for adipocytes (20× magnification). The lower panel shows the relative quantification of Nile red staining of mature oil-filled adipocytes. **(f)** qRT-PCR quantification for AP2, AN, LPL and PPARγ2 adipocytic markers. The expression of each target gene was normalized to GAPDH. Data are presented as mean ± SD from three independent experiments, *n* = 9; ***p < 0.0005.

To confirm the role of NOG in regulating hBMSC differentiation, recombinant NOG (10 ng/ml) was added to the osteoblastic and adipocytic differentiation induction media of hBMSC^+Bone^ cells. NOG-treated hBMSC^+Bone^ cells did not seem to exhibit any changes in cell proliferation (Fig. 7a). Moreover, gene expression analysis revealed downregulation of ACTA2 and TPM1 and upregulation of SERPNB2 expression levels in NOG-treated hBMSC^+Bone^ cells (Fig. 7b). Moreover, NOG treatment diminished the osteoblastic differentiation of hBMSC^+Bone^ cells as demonstrated by an overall reduction in mineralized matrix formation (Fig. 7c), as well as the decreased expression of ALPL, RUNX2 and ON osteoblastic gene markers (Fig. 7d). Furthermore, NOG-treated hBMSC^+Bone^ cells exhibited diminished adipocytic differentiation as evidenced by the reduced number of lipid-filled mature adipocytes (Fig. 7e) and the downregulation of AP2, AN, LPL and PPARg2 adipocytic markers (Fig. 7f). Therefore and in support of the NOG loss-of-function data presented in Fig. 6, recombinant NOG impaired *ex vivo* osteoblastic and adipcytic differentiation of hBMSC^+Bone^ cells.

### BMP4 promotes osteogenic and adipogenic differentiation of hBMSC^−Bone^ cells

**Figure 8:**
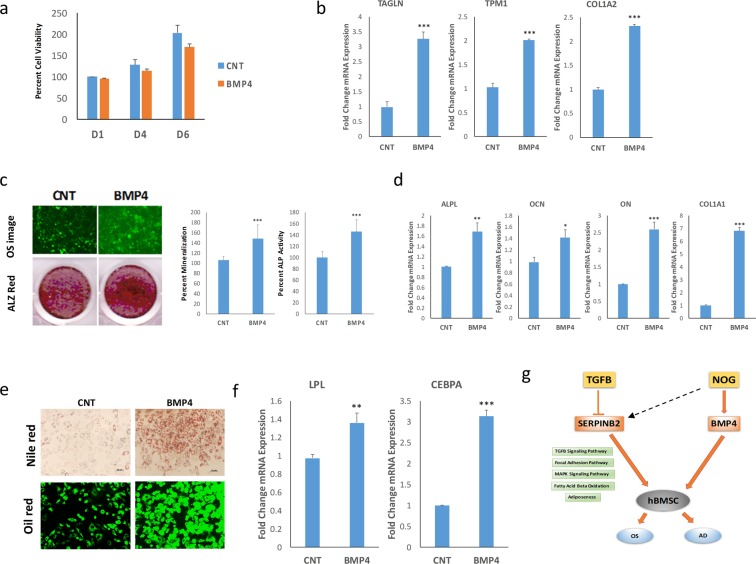
Effect of exogenous BMP4 on osteoblastic and adipocytic differentiation of hBMSC^−Bone^ cells. (**a**) Quantification of cell viability of hBMSC^−Bone^ cells in the presence or absence of recombinant BMP4. **(b)** qRT-PCR quantification for TAGLN, TPM1, and Col1A2 in hBMSC^−Bone^ cells in the presence or absence of recombinant BMP4. The expression of each target gene was normalized to GAPDH. Data are presented as mean ± SD from three independent experiments, *n* = 9; ***p < 0.0005. **(c)** OsteoImage™ staining (20× magnification) of hBMSC^−Bone^ cells which were induced into the osteoblast in the presence or absence of recombinant BMP4. The lower panel shows Alizarin Red S staining. The quantification of mineralized matrix formation for vehicle or recombinant BMP4-treated hBMSC^−Bone^ cells is shown (right panel). Data are presented as relative mean mineralization ± SD from three independent experiments, *n* = 9; *p < 0.0005. **(d)** qRT-PCR quantification of ALPL, OCN, ON, and and COL1A1 osteogenic markers in hBMSC^−Bone^ cells in the presence or absence of recombinant BMP4 under osteogenic induction conditions. The expression of each target gene was normalized to GAPDH. Data are presented as the means ± SD from three independent experiments, *n* = 9;, *p < 0.05**p < 0.005, ***p < 0.0005. **(e)** hBMSC^−Bone^ cells were differentiated into adipocytes for 7 days under the indicated experimental conditions. Upper panel shows fluorescence Nile red staining of mature oil filled adipocytes (20× magnification), whilst the lower panel shows Oil red O staining for adipocytes (20× magnification). The lower panel shows the relative quantification of Nile red staining of mature oil-filled adipocytes. **(f)** qRT-PCR quantification for LPL and CEBPA adipocytic markers. The expression of each target gene was normalized to GAPDH. Data are presented as mean ± SD from three independent experiments, *n* = 9; **p < 0.005, ***p < 0.0005. **(g)** Schematic model illustrating the convergence of BMP and TGFβ in regulating hBMSC differentiation.

BMP4 is one of the BMPs produced by MSCs and plays a role during their osteoblastic differentiation^25^. We observed a significant downregulation of BMP4 gene expression in hBMSC^−Bone^ cells (−9.2 FC) (Fig. 2a). Since NOG antagonizes BMP signaling, we assessed the effects of exogenous BMP4 (50 ng/ml) treatment on hBMSC^−Bone^ cell differentiation. Treatment with BMP4 did not affect the proliferation of hBMSC^−Bone^ cells (Fig. 8a). BMP4 treatment up-regulated TGALN, TPM1, and COL1A2 in hBMSC^−Bone^ cells (Fig. 8b). BMP4-treated hBMSC^−Bone^ cells also exhibited enhanced ALP activity and mineralized matrix formation (Fig. 8c). Concordantly, gene expression analysis showed upregulated ALPL, OCN, ON, and COL1A1 osteoblastic genes (Fig. 8d). Similarly, BMP4-treated hBMSC^−Bone^ cells exhibited enhanced adipocytic differentiation marked by an increased number of lipid-filled mature adipocytes (Fig. 8e) and the increased expression of LPL and CEBPA adipocytic gene markers (Fig. 8f). Theefore, activation of BMP signaling using exogenous BMP4 was able to partially restore the differentiation phenotype of hBMSC^−Bone^ cells.

## Discussion

Delineating signaling pathways regulating hBMSC osteoblastic and adipocytic lineage commitment and differentiation is an area of active investigation. Our recent research highlighted the existence of functional heterogeneity in cultured hBMSCs and the presence of progenitors at different stages of lineage commitment with different functional capacities. Herein we investigated hBMSC^+Bone^ cells, which can differentiate readily into osteoblastic and adipocytic cells versus hBMSC-Bone, which has limited differentiation capacity. Our data revealed TGFβ signaling as a major molecular pathway associated with differentiation responsiveness of hBMSCs. Interestingly, the loss of this signaling pathway in hBMSC^−Bone^ was reversible, suggesting an epigenetic rather than genetic aberration in hBMSC^−Bone^ cells and may be related to cellular heterogeneity of cultured hBMSC.

To gain more in depth insight into the signaling networks associated with the bone and none-bone forming phenotype, we performed global transcriptome profile for both cell types and identified a number of altered signaling pathways. Our data revealed hBMSC^−Bone^ exhibited significant downregulation of several TGFβ responsive genes including TAGLN, TMP1, ACTA2, TGFβ2, SMAD6, SMAD9, BMP2, and BMP4 genes as well as the upregulation of SERPINB2 and NOG. Concordantly, hBMSC^−Bone^ exhibited low basal phosphorylation of the SMAD2 protein, even under induction conditions, suggesting diminished TGFβ and BMP signaling in hBMSC^−Bone^ cells. Activating either TGFβ or BMP signaling in hBMSC^−Bone^ cells was able to partially rescue their differentiation phenotype, thereby implying epigenetic rather than permanent differentiation impairment in those cells.

Our data further unraveled a complex interaction between TGFB and BMP signaling during hBMSC differentiation (Fig. 8g). Exogenous TGFβ1 stimulus exhibited similar effects to those inflicted by SERPINB2 knockdown on restoring the osteogenic and adipogenic differentiation of hBMSC^−Bone^ cells, which would be concordant with our recent finding of bidirectional regulation between SERPINB2 and TGFB signaling^22^. Plasminogen activator inhibitor-2 (also known as PAI-2), is a serine protease inhibitor of the serpin superfamily, which serves as a coagulation factor by inactivating the urokinase plasminogen activator (uPA) and tissue plasminogen activator (tPA)^26^. It is expressed in most cells, especially in macrophages and monocytes, but exists in undetectable quantities in the blood^27^. It is highly expressed during pregnancy, infection, inflammation, and other pathophysiological conditions. Increasing accumulated information on the biochemistry, biology, and clinical aspects of SERPINB2 has revealed its involvement in various intracellular and extracellular physiological and pathological processes^28^. It is involved in maintaining homeostasis during stress, damage, or inflammation^27^. It has been recently reported that SERPINB2 expression is necessary for *in vitro* collagen remodeling in stromal cells^29^. SERPINB2 in stromal cells is a necessary component during extracellular matrix remodeling for fibroblast-contracted collagen 1 matrix formation^29^. Moreover, SERPINB2 was one of the highly regulated genes in hBMSC^−Bone^, suggesting that it most likely plays a role in the blocking of TGFβ-mediated hBMSC differentiation.

On the other hand, the silencing of NOG in hBMSC^−Bone^ has similar effects to those inflicted by an exogenous BMP4 stimulus on promoting osteoblast and adipocytes lineage commitment and differentiation. This suggests that there may well be a plausible convergence of the TGFB and BMP signaling in regulating hBMSC differentiation. BMPs are involved in the TGFβ superfamily, which is known to participate in the regulation of stem cell proliferation and differentiation^30^. Specifically, BMPs are involved in the regulation of osteogenesis and in *in vivo* bone formation^31^. During development, the disruption of BMPs is associated with skeletal and extra-skeletal abnormalities^31,32^. Furthermore, it has been shown that BMPs play an important role in bone healing due to their ability to stimulate the osteoblastic differentiation of hBMSC^33,34^. NOG is a BMP extracellular antagonist that negatively regulates BMP signaling through binding to their receptors leading to impaired osteogenesis and bone formation^35–38^. In our system, exogenous NOG lead to the suppression of BMP signaling, thereby causing impaired *in vitro* bone formation. In addition, overexpression of NOG in the skeletal system leads to reduced bone formation and osteopenia^39,40^. It has been reported that inhibition of NOG either using NOG-neutralizing antibodies or siRNA led to enhanced BMP-dependent osteogenesis of MSC *in vitro* and *in vivo*^41–44^. Interestingly, our data revealed the existence of reciprocal relationship between SERPINB2 and NOG. Therefore, we propose a schematic model illustrating dual signaling network comprising TGFβ-mediated SERPINB and NOG-dependent BMP4 signaling that regulate osteoblastic and adipocytic differentiation of hBMSC-Bone. Our model suggests novel reciprocal relationship between SERPINB2 and NOG.

Our study has some limitations. We have employed human immortalized hBMSC lines in order to dissect the interaction between TGFβ and BMP signaling and in order to avoid confounders of age, gender, *in vitro* replicative senescence phenotype associated with use of primary hBMSC. Also, our studies were based on *in vitro* mechanistic approaches. Future studies examining changes in TGFβ and BMP signaling in cohorts of human subjects of different age and gender as well as its relationship to *in vivo* bone phenotype are needed.

Our study suggests that targeting of the SERPINB/TGFβ and NOG/BMP axes is a plausible future strategy for enhancing *in vitro* osteoblast commitment and differentiation of hBMSC prior to their use in clinical transplantation. Also, the relevance of using small molecules that regulate these signaling pathways in the treatment of patients with impaired bone formation e.g. age-related osteoporosis, remain to be examined in preclinical and clinical studies.

## Supplementary information

Supplementary table 1

Supplementary Table 2

## Data Availability

Raw data will be provided upon acceptance of the manuscript.
