# Author Correction: Convergence of TGFβ and BMP signaling in regulating human bone marrow stromal cell differentiation

**DOI:** 10.1038/s41598-019-54821-8

**Published:** 2019-11-25

**Authors:** Mona Elsafadi, Tasneem Shinwari, Sami Al-Malki, Muthurangan Manikandan, Amer Mahmood, Abdullah Aldahmash, Musaad Alfayez, Moustapha Kassem, Nehad M. Alajez

**Affiliations:** 1 Stem Cell Unit, Department of Anatomy, College of Medicine, King Saud University, Riyadh, Saudi Arabia; 2 College of Agriculture, King Saud University, Riyadh, Saudi Arabia; 3 Prince Naif Health Research Center, King Saud University, Riyadh, 11461 Saudi Arabia; 4 KMEB, Department of Endocrinology, University Hospital of Odense and University of Southern Denmark, Odense, Denmark; 5 Cancer Research Center, Qatar Biomedical Research Institute, Hamad Bin Khalifa University (HBKU), Qatar Foundation, PO Box 34110, Doha, Qatar; 6 College of health & life sciences, Hamad Bin Khalifa University (HBKU), Qatar Foundation, Doha, Qatar

Correction to: *Scientific Reports* 10.1038/s41598-019-41543-0, published online 21 March 2019

The original version of this Article contained a typographical error in the spelling of the author Mona Elsafadi, which was incorrectly given as Mona El-Safadi. This has now been corrected in the PDF and HTML versions of the Article, and in the accompanying Supplementary Information file.

