# Author Correction: Convergence of TGFβ and BMP signaling in regulating human bone marrow stromal cell differentiation

**DOI:** 10.1038/s41598-023-36599-y

**Published:** 2023-07-03

**Authors:** Mona Elsafadi, Tasneem Shinwari, Sami Al-Malki, Muthurangan Manikandan, Amer Mahmood, Abdullah Aldahmash, Musaad Alfayez, Moustapha Kassem, Nehad M. Alajez

**Affiliations:** 1 Stem Cell Unit, Department of Anatomy, College of Medicine, King Saud University, Riyadh, Saudi Arabia; 2 College of Agriculture, King Saud University, Riyadh, Saudi Arabia; 3 Prince Naif Health Research Center, King Saud University, Riyadh, 11461 Saudi Arabia; 4 KMEB, Department of Endocrinology, University Hospital of Odense and University of Southern Denmark, Odense, Denmark; 5 Cancer Research Center, Qatar Biomedical Research Institute, Hamad Bin Khalifa University (HBKU), Qatar Foundation, PO Box 34110, Doha, Qatar; 6 College of Health & Life Sciences, Hamad Bin Khalifa University (HBKU), Qatar Foundation, Doha, Qatar

Correction to: *Scientific Reports* https://doi.org/10.1038/s41598-019-41543-0, published online 21 March 2019

This Article contains errors. In Figure 6C the OS image for SCR siRNA is incorrectly duplicated as the Figure 8C OS image for CNT. The correct Figure 8 and its accompanying legend appear below.

**Figure 8:**
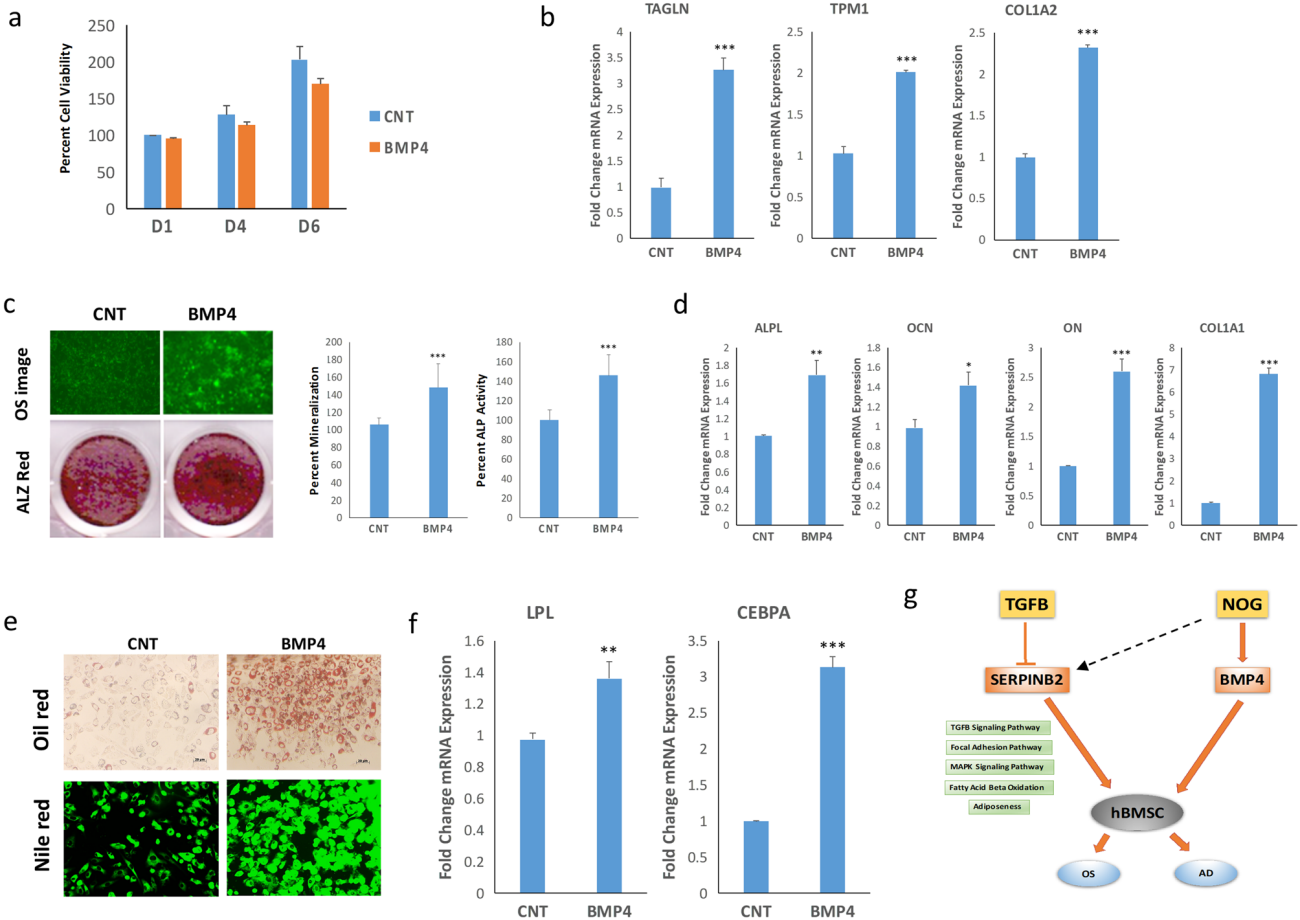
Effect of exogenous BMP4 on osteoblastic and adipocytic differentiation of hBMSC^−Bone^ cells. (**a**) Quantification of cell viability of hBMSC–Bone cells in the presence or absence of recombinant BMP4. (**b**) qRT-PCR quantification for TAGLN, TPM1, and Col1A2 in hBMSC^−Bone^ cells in the presence or absence of recombinant BMP4. The expression of each target gene was normalized to GAPDH. Data are presented as mean ± SD from three independent experiments, *n* = 9; ***p < 0.0005. (**c**) OsteoImage™ staining (20× magnification) of hBMSC^−Bone^ cells which were induced into the osteoblast in the presence or absence of recombinant BMP4. The lower panel shows Alizarin Red S staining. The quantification of mineralized matrix formation for vehicle or recombinant BMP4-treated hBMSC^−Bone^ cells is shown (right panel). Data are presented as relative mean mineralization ± SD from three independent experiments, *n* = 9; *p < 0.0005. (**d**) qRT-PCR quantification of ALPL, OCN, ON, and COL1A1 osteogenic markers in hBMSC^−Bone^ cells in the presence or absence of recombinant BMP4 under osteogenic induction conditions. The expression of each target gene was normalized to GAPDH. Data are presented as the means ± SD from three independent experiments, *n* = 9; *p < 0.05, **p < 0.005, ***p < 0.0005. (**e**) hBMSC^−Bone^ cells were differentiated into adipocytes for 7 days under the indicated experimental conditions. Upper panel shows fluorescence Nile red staining of mature oil filled adipocytes (20× magnification), whilst the lower panel shows Oil red O staining for adipocytes (20× magnification). The lower panel shows the relative quantification of Nile red staining of mature oil-filled adipocytes. (**f**) qRT-PCR quantification for LPL and CEBPA adipocytic markers. The expression of each target gene was normalized to GAPDH. Data are presented as mean ± SD from three independent experiments, *n* = 9; **p < 0.005, ***p < 0.0005. (**g**) Schematic model illustrating the convergence of BMP and TGFβ in regulating hBMSC differentiation.

