# Supplementary material for: Convergence of TGFβ and BMP signaling in regulating human bone marrow stromal cell differentiation
Source: Sci Rep. 2019 Mar 21;9:4977. doi: 10.1038/s41598-019-41543-0 (PMC6428815; doi:10.1038/s41598-019-41543-0)
Supplement: Supplementary file 1 — Supplementary table 1 [file 41598_2019_41543_MOESM1_ESM.docx]

**Convergence of TGFβ and BMP signaling in regulating human bone marrow**

**stromal cells differentiation**

Mona Elsafadi, Tasneem Shinwari, Sami Al-Malki, Muthurangan Manikandan, Amer Mahmood, Abdullah Aldahmash, Musaad Alfayez, Moustapha Kassem, Nehad M. Alajez

**Supplementary Table 1: Real-Time PCR primer sequences used in this study**

**A: SYBR Green Primers**

| **Gene Name** | **Forward Primer (5´–3´)** | **Reverse Primer (5´–3´)** |
| --- | --- | --- |
| GAPDH | CTGGTAAAGTGGATATTGTTGCCAT | TGGAATCATATTGGAACATGTAAACC |
| Runx2 | CACCATGTCAGCAAAACTTCTT | ACCTTTGCTGGACTCTGCAC |
| ALPL | GACGGACCCTCGCCAGTGCT | AATCGACGTGGGTGGGAGGGG |
| OCN | GGCAGCGAGGTAGTGAAGAG | CTCACACACCTCCCTCCTG |
| PPARG | TTCTCCTATTGACCCAGAAAGC | CTCCACTTTGATTGCACTTTGG |
| AP2 | GCCAGGAATTTGACGAAG TC | TGGTTGATTTTCCATCCCAT |
| LPL | CTTGGAGATGTGGACCAGC | GTGCCATACAGAGAAATCTC |
| ADIPOQ | GCAGTCTGTGGTTCTGATTCCATAC | GCCCTTGAGTCGTGGTTTCC |
| COL1A1 | TGACGAGACCAAGAACTG | CCATCCAAACCACTGAAACC |
| BMP4 | \| TGAGTGCCATCTCCATGCTGTA \| \| --- \| | CGGCACCCACATCCCTCTACTA |
| OPN | cagttcagaagaggagg | tcagcctcagagtcttcatc |
| ON | GAGGAAACCGAAGAGGAGG | GGGGTGTTGTTCTCATCCAG |
| CEBPA | TATAGGCTGGGCTTCCCCTT | AGCTTTCTGGTGTGACTCGG |

**B: Taqman Real-Time PCR Primers**

| **Gene Name** | **Assay ID** | **Cat No.** |
| --- | --- | --- |
| TAGLN | Hs01038777_g1 | 4331182 |
| GAPDH | Hs02758991_g1 | 4331182 |
| ACTA2 | Hs00426835_g1 | 4331182 |
| SERPINB2 | Hs01010736_m1 | 4331182 |
| TPM1 | Hs00165966_m1 | 4331182 |
| COL1A2 | Hs01028972_m1 | 4351372 |
| SmAD2 | Hs00998187_m1 | 4331182 |
| SMAD4 | Hs00929647_m1 | 4331182 |
